# Glycated Casein by TGase-Type Exerts Protection Potential against DSS-Induced Colitis via Inhibiting TLR4/NF-κB Signaling Pathways in C57BL/6J Mice

**DOI:** 10.3390/foods12183431

**Published:** 2023-09-14

**Authors:** Hui Chu, Weiling Liu, Cong Zhao, Tong Yin, Jia Shi, Wei Zhang

**Affiliations:** 1 Key Laboratory of Dairy Science, Ministry of Education, Northeast Agricultural University, Harbin 150030, China; 2 Department of Food Science, Northeast Agricultural University, Harbin 150030, China

**Keywords:** glycated casein, transglutaminase, anti-inflammatory effect, TLR4/NF-kB pathway

## Abstract

Glycation by transglutaminase (TGase)-type could effectively improve the structure and functional properties of proteins. However, the influence on intestinal inflammation or the underlying mechanisms has not been investigated. The goal of this research was to compare the bioactivities between glycated casein generated from the TGase-catalyzed reaction and oligochitosan as well as casein using a mouse model of dextran sulfate sodium (DSS)-induced intestinal inflammation to examine the protective effects and the underlying mechanism of glycated casein on intestinal inflammation. Eight groups of C57BL/6 mice were randomly assigned in this study: Control group: standard diet for 35 days; Model group: standard diet for 28 days and then colitis induction; Pretreated groups: different levels (200, 400, 800 mg/kg BW) of casein or glycated casein for 28 days before colitis induction. The mice were drinking water containing a 3% DSS solution for seven days of mice to cause colitis. The results indicated that glycated casein and casein at 200–800 mg/kg BW all relieved DSS-induced weight loss, reduced disease activity index (DAI) score, alleviated colon length shortening, weakened the destruction of colonic mucosal structure, decreased serum LPS, and MPO, IL-1β, IL-6 and TNF-α levels in serum and colon, as well as regulated the expression of proteins involved in the TLR4/NF-κB signaling pathway in a concentration-dependent manner. Glycated caseinate showed a better protective effect against DSS-induced colitis than casein, highlighting that the TGase-type glycation of proteins as a potential functional food ingredient might be a helpful method for gut health.

## 1. Introduction

Chronic nonspecific colitis known as ulcerative colitis is characterized by occult hemorrhagic diarrhea, cryptic structures degradation, and broad mucosal inflammation [1]. The complex interaction of genetic and environmental factors including dietary changes or food contaminants, gut microbiota imbalance, immunological problems, and oxidative stress all lead to ulcerative colitis [2]. Serious side effects of untreated ulcerative colitis include peritonitis and colon cancer [3]. Ulcerative colitis causes reduced microbial diversity and an increased abundance of pathogenic bacteria, which can reflect dysbiosis of the intestinal flora. Therefore, regulating the balance of the intestinal flora may be an effective way to modulate the symptoms of ulcerative colitis. Dextran sulfate sodium (DSS) is widely employed to establish the mouse colitis model, which closely mimics human ulcerative colitis with symptoms including weight loss, diarrhea, and colonic shortening [4]. Serious problems include thrombosis, toxic megacolon, as well as colorectal cancer, which can eventually result from ulcerative colitis [5]. As the prevalence of ulcerative colitis continues to rise each year, it has become an urgent global public health issue [6].

Currently, some dietary components (such as protein, polysaccharides, dietary fiber, phenols, and probiotics) have been used to prevent and alleviate colitis [7]. The oligomannuronate from alginate oligosaccharide improved the symptoms of colitis and ameliorated the gut barrier function in vivo [8]. Fish skin collagen hydrolysate attenuated barrier dysfunction by TNF-α and improved the clinical signs of colitis by DSS [9]. In recent years, several modifications of proteins such as the Maillard reaction have been used to enhance functional properties and bioactivities of proteins [10]. The Maillard reaction products of ovalbumin ameliorated DSS-induced colitis symptoms and restored intestine integrity [11]. However, the Maillard reaction exhibits many drawbacks; for example, the reaction conditions display high requirements and are easily affected by the external environment. During the reaction process, side reactions may occur, which affects the efficiency of the reaction. Moreover, the structure and properties of the products obtained from the reaction cannot be completely controlled, thereby forming toxic and mutagenic substances and resulting in unliked colors as well as nutritional disorders [12]. TGase-type glycation of proteins is another approach that could also bring about functionality and bioactivity changes. Oligochitosan–glycated ovalbumin by TGase enhances the functional properties of ovalbumin [13]. Glycated casein via TGase-type increases the immune-promoting activity of casein [14]. However, the potential effects of glycated proteins by TGase-type on DSS-induced colitis have not been studied so far.

Herein, the DSS-induced colitis in mice was established to explore the protective effects and related mechanisms of glycated caseinate, focusing on alterations in the biochemical markers from serum and colon, and the expression of key proteins involved in the TLR4/NF-κB signaling pathway. The protective effects of glycated casein and casein were compared based on the fact that oligochitosan glycation by TGase could impact the effects of casein. Our results provide evidence for the potential effect of glycated casein as a functional ingredient for the dietary therapy of ulcerative colitis.

## 2. Materials and Methods

### 2.1. Materials and Reagents

Casein (protein content of 984.2 g/kg) was bought from Sigma-Aldrich Co. (St. Louis, MO, USA). Oligochitosan was purchased from Zhejiang Golden Shell Biochemical Co. (Hangzhou, China). Transglutaminase (TGase) with the enzyme activity of 147 units (U)/g was provided by Jiangsu Yiming Fine Chemical Industry CO., Ltd. (Taixing, China). DSS (Mw 35–50 kDa) was obtained from MP Biomedicals (Santa Ana, CA, USA). ELISA kits of lipopolysaccharide (LPS), tumor necrosis factor-α (TNF-α), interleukin (IL)-1β, IL-6 as well as myeloperoxidase (MPO) were bought from Nanjing Jiancheng Bioengineering Institute (Nanjing, China). Hematoxylin and eosin (H&E) were obtained from Beyotime Co., Ltd. (Shanghai, China). Primary antibodies (TLR4, NF-κB, IKB-α, MyD88) were purchased from Abcam PLC (Cambridge, UK). β-Actin was provided by ABclonal Technology Co., Ltd. (Wuhan, China).

### 2.2. Preparation of Glycated Casein by TGase-Type

The glycated casein by TGase-type was fabricated as previously described [15]. Briefly, oligochitosan was added to the casein solution (80 g/L) to reach a 1:3 ratio of acyl donor and oligochitosan acceptor, and then TGase (10 U/g) was mixed with the solutions to carry out a glycation reaction at 37 °C for 3 h. The mixture solution was treated at 85 °C for 5 min, and then rapidly cooled to room temperature; two isoelectric washings were performed, and then neutralized to pH 7.0 to prepare the glycated casein by TGase-type.

### 2.3. Animals and Experimental Design

**Figure 1:**
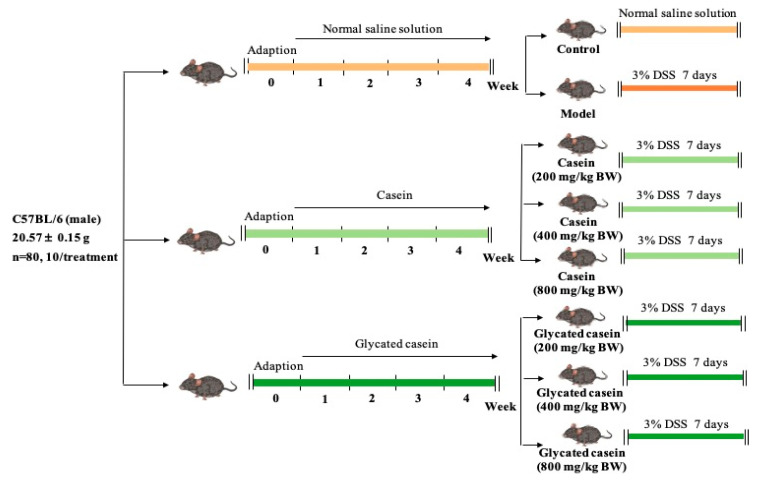
Schematic overview of treatments in DSS-treated mice and untreated mice.

From Beijing Vital River Experimental Animal Technical Co., Ltd. (Beijing, China), Eighty male C57BL/6 mice were obtained. This experiment was carried out according to the Guidelines for the Animal Care and Use Committee of Northeast Agricultural University and approved by the animal experiments of the Animal Ethics Committee of Northeast Agricultural University (Approval number: NEAUEC2022031). All of the mice (8 weeks old) were housed in typical cages with a consistent 12 h dark/light cycle, a humidity of 40–60%, and a temperature of 22 ± 1 °C. The experimental design is displayed in Figure 1. Male mice with average body weights of 20.57 ± 0.15 g were selected and randomly divided into 8 groups (*n* = 10) after 7 days of adaptation. The control group was fed a normal saline solution (0.9%) of mice for 35 days. In the model group, the mice were given a normal saline solution for 28 days, and then drank water containing 3% DSS for 7 days. In the 6 treatment groups, the mice were fed different levels (200, 400, 800 mg/kg BW) of casein or glycated casein samples for 28 days, and the 3% DSS solution was then added to drinking water for 7 days. Finally, cervical dislocation was used to end the lives of all mice. The blood samples were rapidly prepared from the eyeballs. In addition, the colon samples in mice were preserved at −80 °C for further experiments after freezing in liquid nitrogen.

### 2.4. Determination of Body Weight, Colon Length, and Disease Activity Index

Every day, the mice body weights were checked. The colonic lengths of the mice were evaluated. Disease activity index (DAI), which is determined as the sum of overall health status, weight loss, stool consistency, and degree of fecal hemorrhage, was used to assess the severity of DSS-induced colitis in mice. The DAI of mice was noted daily. The score standards were as described previously [16].

### 2.5. Histological Examination

The colonic tissue samples of each animal were histologically observed. The colon samples in experimental groups were fixed in 4% paraformaldehyde for 24 h, and then were embedded in paraffin. Tissue examinations were performed in 4 μm sections; hematoxylin and eosin (H&E) staining and analysis by microscopy were conducted. The histological score was referred to in a previous report [17]. The score standards were calculated as the sum of five factors including inflammation, extent, regeneration, crypt damage, and percent involvement.

### 2.6. Biochemical Marker Analysis

The colon samples were rinsed in physiological saline and then homogenized with a buffer solution at 4 °C. LPS in serum was evaluated by ELISA kits. The levels of MPO activity and the inflammation cytokines for IL-1β, IL-6, and TNF-α in serum and colon were measured by ELISA kits.

### 2.7. Western Blot

The colon samples (30 mg) were grinded with liquid nitrogen. According to the kit’s instructions, the total protein content was obtained, combined with the loading buffer solution (4:1), and heated in a boiling water bath for 5 min. The same amount of protein (40 µg) was separated on SDS-PAGE gels, and the target protein obtained by electrophoresis was transferred to the PVDF membrane. This membrane was then sealed with a 5% skimmed milk solution for 120 min, rinsed twice with PBST for 5 min, as well as incubated with the primary antibody working solution at 4 °C overnight. The film was washed three times with PBST, and then the secondary antibody (1:2000 dilution) was applied. A reagent for enhanced chemiluminescence reagent was used to find the signals. The detected proteins were TLR4 (1:500 dilution), NF-κB p65 (1:1000 dilution), IKB-α (1:1000 dilution), and MyD88 (1:1000 dilution), respectively. β-actin (1:2000 dilution) was as an internal reference.

### 2.8. Statistical Analysis

Data analysis was performed using GraphPad Prism 7.0 (GraphPad, La Jolla, CA, USA). All experimental data were presented as the mean ± standard deviations (S.D.). One-way analysis of variance (ANOVA) and Dunnett’s multiple comparison tests were used for statistical analysis. Statistical significance was fixed at * *p* < 0.05.

## 3. Results

### 3.1. Glycated Casein by TGase-Type Improved Colitis Symptoms

**Figure 2:**
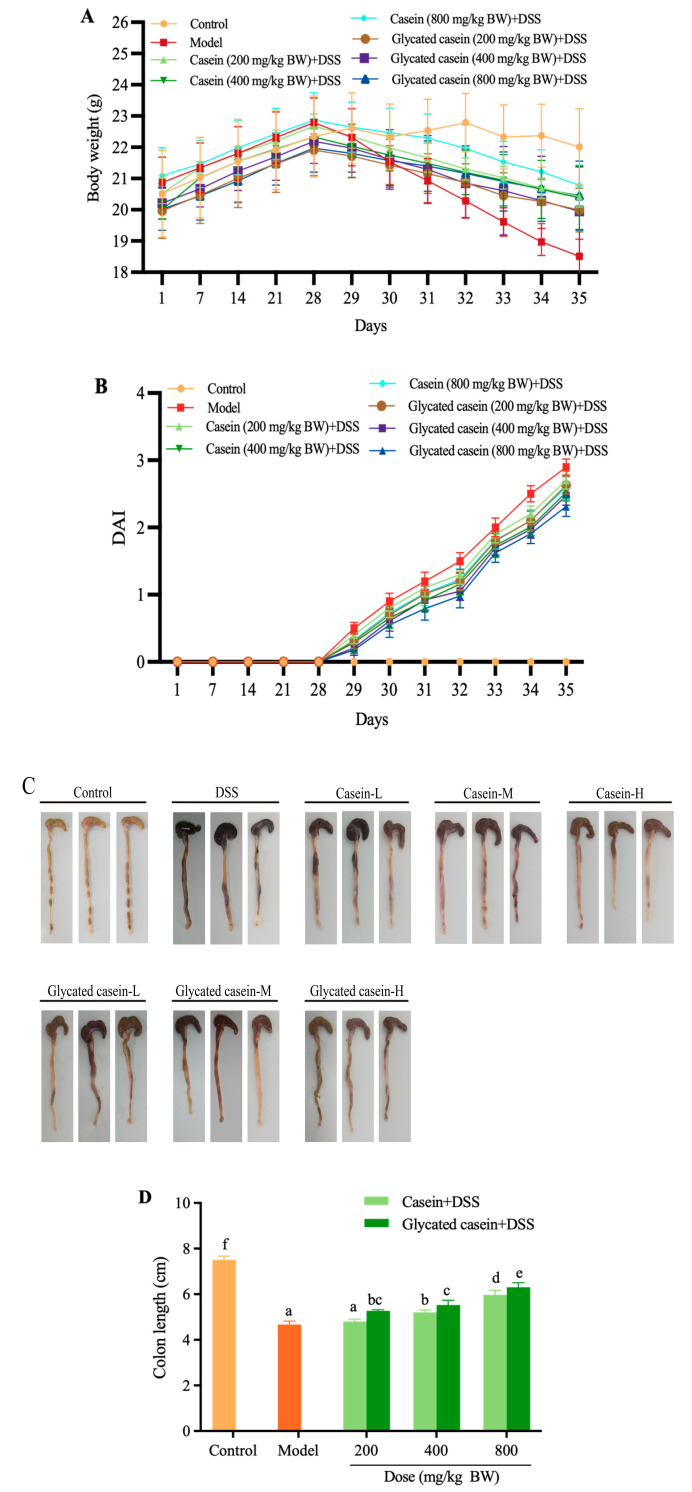
Body weight (**A**), DAI (**B**), and colonic length (**C**,**D**) of each group during mouse model development. Different lowercase letters above the columns indicate that the mean values differed (*p* < 0.05).

The glycated casein by TGase-type had a glucosamine content of approximately 5.73 g/kg protein, indicating that oligochitosan was conjugated covalently into casein molecules. The body weight of all mice first increased before 28 days, and then remarkably reduced after DSS induction (Figure 2A). However, glycated casein and casein treatment could prevent weight loss when exposed to DSS. On Day 35, DAI in mice with DSS challenges was 2.90. Pretreatments with glycated casein and casein caused a sharp reduction in DAI, which was 2.47–2.70 and 2.31–2.60, respectively (Figure 2B). In comparison to that in the DSS treatment group, DAI decreased by 6.90–14.83% and 10.34–20.34% in glycated-casein- and casein-treated mice, respectively. Compared with the control mice, the colon length of the DSS treatment group was dramatically reduced. The colon lengths of glycated casein and casein were 1.13–1.35 and 1.03–1.28 times greater than those of mice in the model group (*p* < 0.05) (Figure 2C,D). This result indicated that both glycated casein by TGase-type and casein could improve colitis symptoms. Interestingly, glycated casein by TGase-type was more effective against colitis compared to casein.

### 3.2. Glycated Casein by TGase-Type Alleviated Colon Injury in Mice against DSS-Induced Colitis

**Figure 3:**
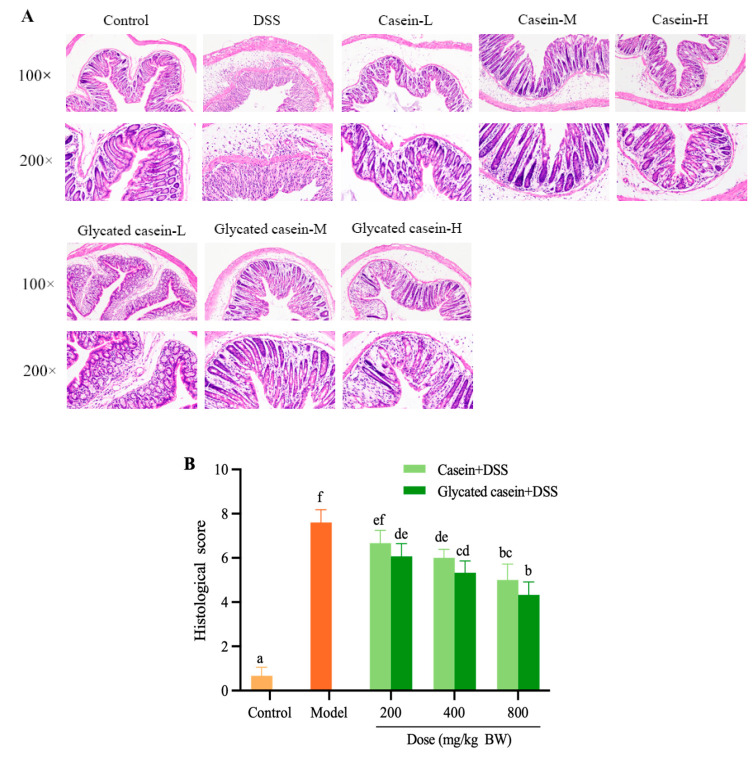
Effect of glycated casein and casein on the histopathological analysis for colon. (**A**) H&E staining of colon tissue samples at 100× and 200× magnification; (**B**) Histological score of colon tissue samples. Different lowercase letters above the columns indicate that the mean values differed (*p* < 0.05).

Colon damage was measured by H&E staining (Figure 3A). The morphology of colonic mucosa presented without inflammation in the mice of the control group, which had intact mucosal, submucosal as well as muscular layers. Moreover, the mucosal layer showed properly arranged crypts and an amount of goblet cells. Meanwhile, the mice of the DSS treatment group exhibited the typical symptoms of colitis, including damaged colonic mucosa, goblet cell exhaustion, crypt disruption, submucosa inflammatory cell infiltration, as well as muscle layer edema and thickening. However, pretreatment with casein and glycated casein by TGase-type could alleviate the pathological damage. As shown in Figure 3B, the score of colon tissue of mice in the control group was only 18% of those in the DSS treatment group. Moreover, in comparison to that of the DSS group, the colon tissue injury score in mice treated with casein and glycated casein dose-dependently decreased by 12.28–34.21% and 20.17–42.99%, respectively. Especially compared with casein, glycated casein exhibited better protective effects on the colon, specifically in maintaining intact crypts and decreasing goblet cells loss. Therefore, glycated casein by TGase-type alleviated the pathological lesion of the colon induced by DSS.

### 3.3. Glycated Casein by TGase-Type Reduced Systemic Inflammation in DSS-Induced Mice

**Figure 4:**
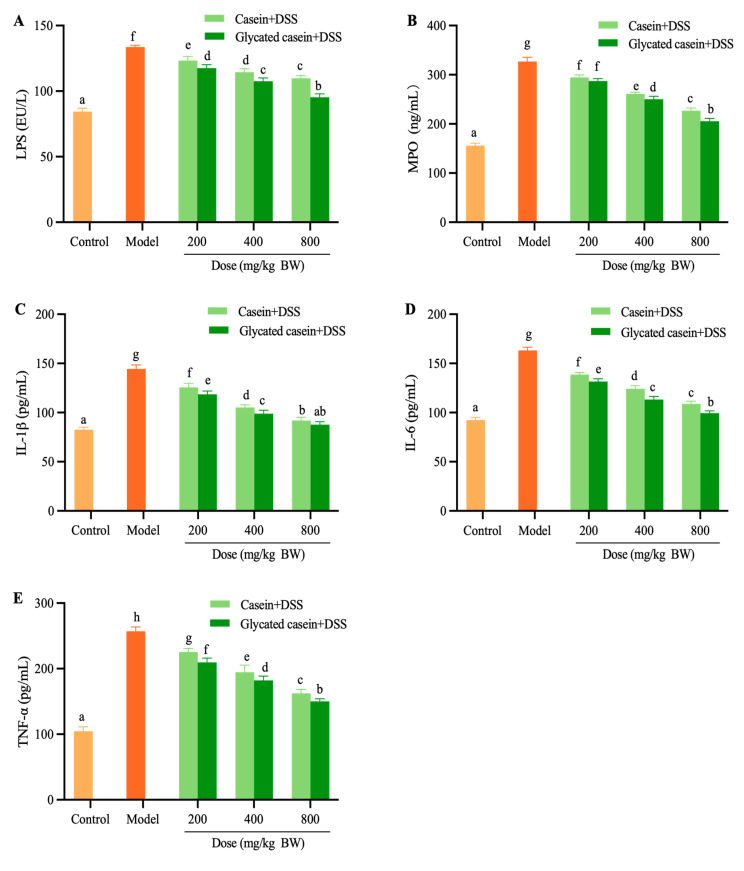
Effect of glycated casein and casein on the LPS (**A**), MPO (**B**), IL-1β (**C**), IL-6 (**D**), and TNF-α (**E**) in serum. Different lowercase letters above the columns indicate that the mean values differed (*p* < 0.05).

As demonstrated in Figure 4, DSS-challenged mice showed the highest LPS level in serum (133.69 EU/L), while the levels of mice in glycated-casein by TGase-type and casein treatment significantly decreased by 12.08–28.67% and 8.55–17.82%, respectively. The serum levels of MPO of glycated-casein- and casein-treated mice decreased by 12.19–37.16% and 9.88–30.60%, respectively, in comparison to the DSS group. The serum concentrations of pro-inflammatory cytokines (IL-1β, IL-6, and TNF-α) were significantly enhanced in the model group when compared to the control group. Briefly, the levels of IL-1β, IL-6, and TNF-α in mice of the glycated casein group significantly decreased by 17.89–39.27%, 19.35–39.11%, and 18.42–41.63%, respectively. The mice in the casein treatment group also exhibited reduced serum IL-1β, IL-6, and TNF-α levels by 13.09–36.37%, 14.98–33.29%, and 12.32–36.84% in a concentration-dependent manner, respectively. When compared to the casein treatment group, the glycated casein treatment group presented remarkably inhibited serum levels of pro-inflammatory cytokines caused by DSS-induced colitis. In summary, the effects of glycated casein by TGase-type intervention on systemic inflammation may play an important role in the mitigation of DSS-induced colitis.

### 3.4. Glycated Casein Relieved Colonic Inflammation in DSS-Induced Mice

**Figure 5:**
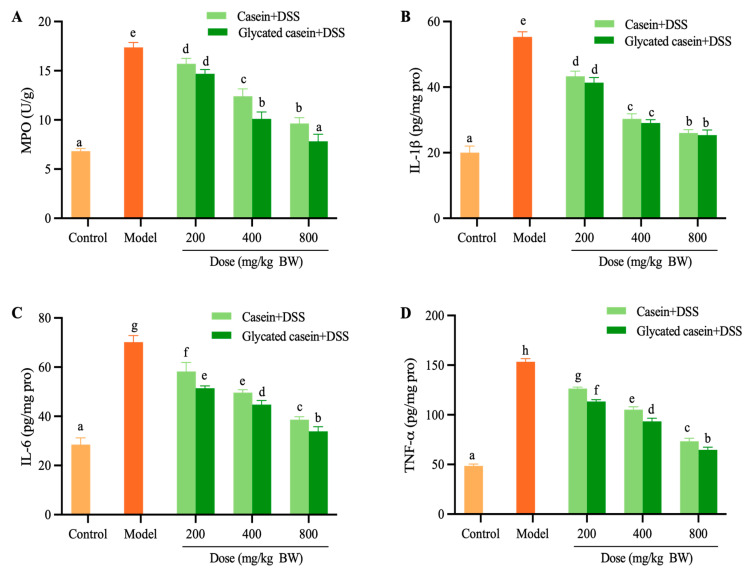
Effect of glycated casein and casein on the MPO (**A**), IL-1β (**B**), IL-6 (**C**), and TNF-α (**D**) in the colon. Different lowercase letters above the columns indicate that the mean values differed (*p* < 0.05).

The MPO activity associated with neutrophil infiltration in colon tissue is a key marker of inflammation. As shown in Figure 5, MPO activity in colon tissues increased after DSS exposure, whereas glycated casein and casein dramatically reduced their upregulations. Briefly, the activity of colonic MPO in glycated casein treatment at the dose of 200–800 mg/kg BW decreased by 15.37–54.89%. Casein treatment could reduce the activity of MPO by 9.59–44.45%. DSS exposure increased the concentration of IL-1β, IL-6, and TNF-α, while glycated casein and casein treatment significantly decreased the three pro-inflammatory cytokines (*p* < 0.05). When compared to the DSS group, the levels of IL-1β, IL-6, and TNF-α in the casein group reduced by 21.69–53.01%, 25.53–44.99%, 17.54–52.14%, whereas glycated casein brought about a decline of 25.30–54.22%, 26.70–51.70%, and 26.03–57.75%, respectively. Notably, glycated casein treatment exhibited a more effective anti-inflammatory effect of DSS-induced colitis. The results revealed that glycation by TGase can enhance the effect of glycated casein on DSS-induced colitis.

### 3.5. Glycated Casein Inhibited TLR4/ MyD88/NF-kB Signaling in Colon

**Figure 6:**
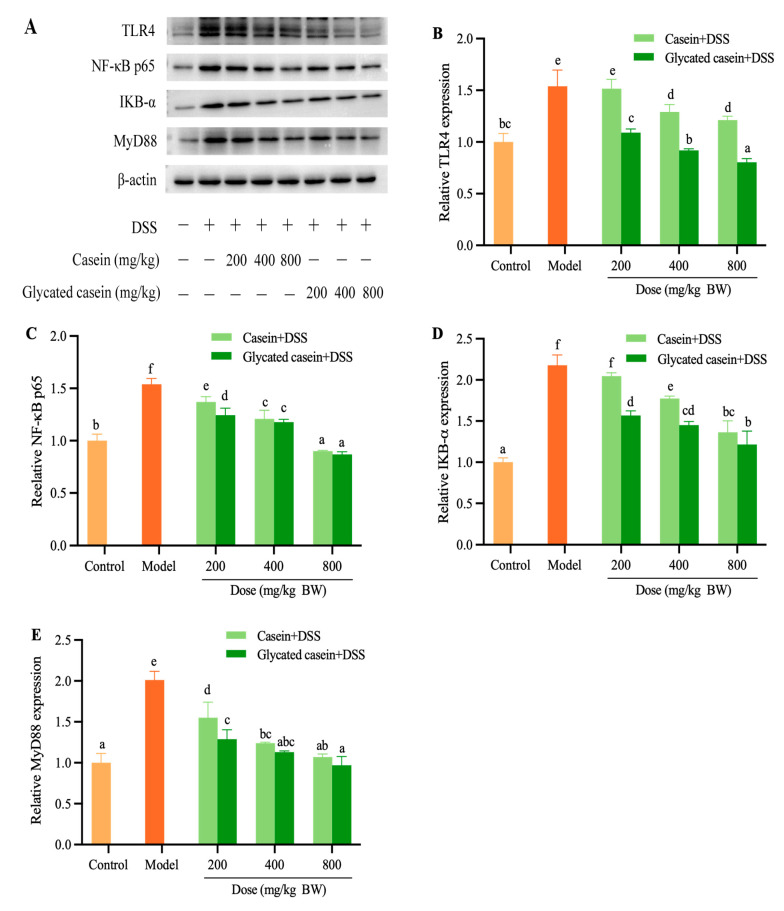
The expression levels of TLR4, NF-κB p65, IKB-α and MyD88 in the colon (**A**–**E**). Different lowercase letters above the columns indicate that the mean values differed (*p* < 0.05).

The expression of proteins (TLR4, NF-κB p65, IKB-α, and MyD88) was further analyzed by Western blot assays. The protein expression of TLR4, MyD88, IKB-α, and NF-kB p65 in the model mice group demonstrated a significant increase when compared to the control group (*p* < 0.05), which is shown in Figure 6. The control group was defined with relative protein expression levels (i.e., 1.00-fold) for four proteins. For the result of DSS treatment, the mice in the model group were detected with downregulated protein expression levels for TLR4, MyD88, IKB-α, and NF-kB p65, which corresponded to 1.54-, 2.01-, 2.18-, and 1.54-fold decrease, respectively. Glycated casein intervention significantly downregulated the expression of TLR4, MyD88, IKB-α, and NF-kB p65, which reached 0.80–1.09-, 0.97–1.29-, 1.21–1.57-, and 0.87–1.24-fold decrease, respectively, while casein treatment decreased four key protein expressions by 1.21–1.51-, 1.07–1.55-, 1.36–2.05-, and 0.90–1.37-fold, respectively. In summary, these results preliminarily demonstrated that both casein and glycated casein could significantly decrease the expression of key proteins. Glycated casein by TGase-type had a beneficial protective effect on DSS-induced colitis.

## 4. Discussion

Ulcerative colitis is a type of inflammatory bowel disease which is a chronic nonspecific colorectal inflammation [18]. It has a complicated multifactorial pathogenesis that includes immune response disorders, genetic susceptibility, environmental variables, as well as changes in gut microbiota [19,20]. In recent years, ulcerative colitis treatments mainly pay attention to the anti-inflammatory effects and immunological regulatory activities, while they frequently have unfavorable side effects. The inflammatory mechanisms of colitis are studied using a DSS-induced colitis model [21]. Currently, many studies find that food-assisted therapy has a beneficial effect on the treatment of inflammatory bowel disease, such as dietary polyphenols, proteins, dietary fiber, prebiotics, etc. [22]. The major fatty acid in royal jelly, 10-hydroxy-2-decenoic acid, improves the effects of DSS-induced colitis by mediating the NLRP3 inflammasome-mediated pyroptosis pathway and increasing intestinal barrier function [23]. Administration of pectic polysaccharides relieves DSS-induced colitis in alleviating tissue damage and suppressing the production of pro-inflammatory cytokines, as well as maybe acting as a therapeutic strategy in the management of gut inflammation [24]. Green tea extract containing piper retrofractum fruit inhibits DSS-induced colonic inflammation by reducing the expression of miR-21 and NF-κB [25]. Collagen peptide promotes DSS-induced colitis by disrupting the gut microbiota and controlling macrophage polarization [26]. The Maillard product of ovalbumin blocks the release of lysine and essential amino acids, as well as improves the gut microbiota and levels of short-chain fatty acids in vivo [11]. Except for the Maillard reaction, the enzymatic method can be used for glycated proteins. TGase along with amino-containing saccharides is another way to obtain glycated proteins because TGase can conjugate amino-containing saccharides into proteins.

Previously, the glycation of proteins by TGase-type has been extensively studied for their functional properties. It has been reported that TGase-catalyzed glycation enhanced the physicochemical properties and functionalities of *Lentinus edodes* protein fraction [27]. The glycated casein and soybean protein under the action of glucosamine and TGase exhibit stronger interfacial and rheological properties; however, glycated casein and soybean by oligochitosan and TGase show better gelation, emulsion stability, as well as water-binding properties [28,29]. Chitosan oligosaccharides and TGase-induced glycation of glutamine have the potential to be an effective treatment method to prevent the formation of advanced glycation end products in fishery products [30]. Glycation via TGase and glucosamine of β-lactoglobulin can reduce the antigenicity and allergenicity [31]. In our previous work, the changes in structure caused the different functionalities and bioactivities. It was found that glycated casein hydrolysate by TGase-type was more effective than casein hydrolysate in improving the barrier function or relieving the acrylamide-treated barrier dysfunction of the IEC-6 cells [32]. This indicates that protein glycation by the TGase-type has the potential to enhance protein bioactivity to maintain intestinal health. However, whether glycated casein and casein have different efficiencies on colitis and the underlying mechanisms remains unclear.

In this study, TGase-type glycation was used to obtain glycated casein and assess the protective effect on DSS-induced colitis. The results demonstrated that both glycated casein and casein administration significantly attenuated DSS-induced colitis in mice by alleviating endotoxemia, reducing systematic and colonic inflammation, decreasing neutrophil-derived MPO activity, as well as regulating the TLR4/NF-kB signaling pathway. Notably, compared to casein, glycated casein was more effective against colitis. The inflammatory responses are considered one of the important pathological mechanisms that induce colitis. A stable internal environment is maintained by the intestinal mucosa, an essential barrier within the intestine that prevents harmful substances from the intestine, such as bacteria and toxins, from crossing the intestinal mucosa and entering other tissues, organs, and the bloodstream [18]. In this study, the colonic tissues were examined using H&E staining. According to our research, intestinal villi in mice with DSS-induced colitis were scarce and disorganized, and there was a large infiltration of inflammatory cells, which indicated that DSS severely disrupted the intestinal structure. Interestingly, glycated casein by TGase-type treatment partly improved the intestinal injury. MPO is a glycoprotein released by neutrophils upon stimulation [33]. After neutrophils are activated, they are released into extracellular or phagocytic bodies to accelerate local intestinal inflammation [34]. In this study, we found that the glycated casein by TGase-type group presented remarkably inhibited levels of pro-inflammatory cytokines and MPO activity in serum and colon caused by DSS-induced colitis compared to the casein group. Collectively, the effects of glycated casein by TGase-type intervention on systemic and colonic inflammation may play a key role in the mitigation of DSS-induced colitis.

**Figure 7:**
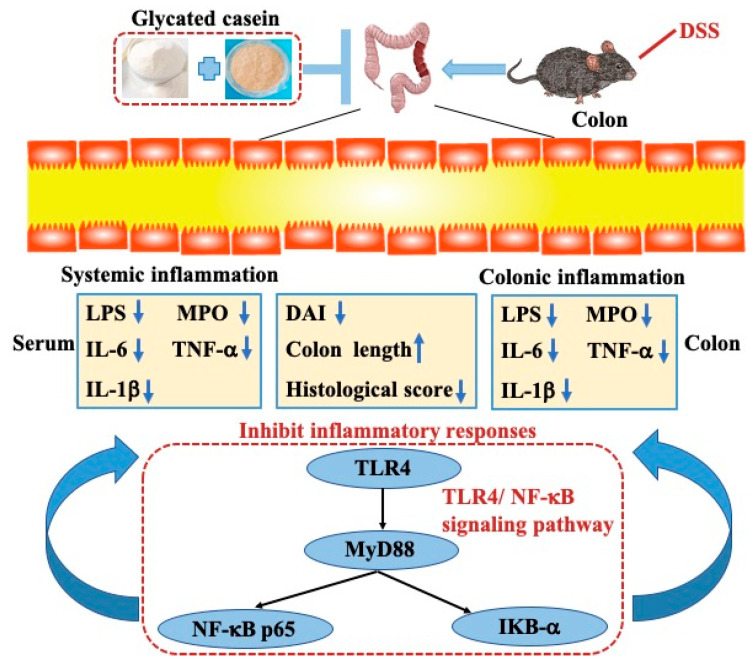
The protective mechanism of glycated casein by TGase-type against DSS-induced colitis.

The possible molecular mechanisms were further analyzed based on these findings. Many studies have confirmed various signaling pathways related to inflammatory responses [35]. The TLR4/NF-kB signaling pathway is one of the important regulatory mechanisms for inflammation [36]. It has been well established that TLR4 and the downstream binding protein of MyD88 facilitate DSS-induced colitis by activating NF-kB to promote the transcription of inflammation genes [37]. In a DSS mouse model, over-expression of TLR4 has been found to enhance inflammatory responses to mucosal injury and promote colitis-induced tumorigenesis [38]. In this study, the results from Western blot assays suggested that higher levels of TLR4, NF-κB p65, IKB-α and MyD88 were detected in the DSS-induced colitis compared to the control group. Glycated casein down-regulated the expression of the key proteins (TLR4, NF-κB p65, IKB-α and MyD88). Therefore, glycated casein by TGase-type potentially inhibited the activation of the TLR4/NF-κB signaling pathway and mitigated the release of inflammatory cytokines (Figure 7). The chemical structure and compositions of glycated protein and protein are different, and these differences in chemical properties have certain impacts on their activity [31,39]. In our previous study, we used LC-MS/MS analysis to identify oligochitosan binding sites at the Gln residues of αS1-casein and αS2-casein [40]. The LC-MS/MS analysis results are also directly supported by the chemical modification mechanism of the glycation by TGase-type. The oligochitosan bound in casein may partially or completely contribute to the change in biological activity.

The need for high-quality protein components in the food industry is rising as the world’s population and health consciousness rise. In the food industry, casein, a well-known component of milk proteins, has long been used. The bioactivities and functionalities of proteins may be improved using TGase-mediated oligochitosan glycation of proteins as a promising technique. These findings in the current study offer new insights into the protective mechanisms of action of bioactive compounds from casein and oligochitosan against colitis. This study also highlighted the possible application of the TGase-type glycation of proteins in the food industry and demonstrated the advantages of glycated protein via TGase-type for health. Glycated casein may be used as a functional protein ingredient or food supplement with increased health benefits for the body, particularly in alleviating intestinal inflammation.

## 5. Conclusions

In summary, the present study first found that glycated casein by TGase-type alleviated DSS-induced colitis in mice. The results showed that glycated casein by TGase-type and casein could both slow DSS-induced weight loss, decrease DAI score, increase colon length, alleviate the destruction of the colonic structure, as well as decrease serum LPS, and MPO, IL-1b, IL-6, and TNF-a levels in serum and colon. In addition, the administration of glycated casein by TGase-type and casein regulated the expression of key proteins (TLR4, NF-κB p65, IKB-α, and MyD88) in a concentration-dependent manner. Moreover, glycated casein by TGase-type and casein inhibited the activation of the TLR4/NF-κB signaling pathway as a protective mechanism against DSS-induced colitis. Compared with casein samples, glycated casein by TGase-type exhibited more effectiveness with significantly improved indices against DSS-induced colitis. Our work provided clear evidence of the biological activity of glycated casein by TGase-type, which could be a potential functional food ingredient for gut health.

## Data Availability

All data are contained within the article.
